# Non-Conserved Residues in *Clostridium acetobutylicum* tRNA^Ala^ Contribute to tRNA Tuning for Efficient Antitermination of the *alaS* T Box Riboswitch

**DOI:** 10.3390/life5041567

**Published:** 2015-09-28

**Authors:** Liang-Chun Liu, Frank J. Grundy, Tina M. Henkin

**Affiliations:** Department of Microbiology and Center for RNA Biology, The Ohio State University, Columbus, OH 43210, USA; liu.770@buckeyemail.osu.edu (L.-C.L.); grundy.5@osu.edu (F.J.G.)

**Keywords:** T box, tRNA, antitermination, riboswitch

## Abstract

The T box riboswitch regulates expression of amino acid-related genes in Gram-positive bacteria by monitoring the aminoacylation status of a specific tRNA, the binding of which affects the folding of the riboswitch into mutually exclusive terminator or antiterminator structures. Two main pairing interactions between the tRNA and the leader RNA have been demonstrated to be necessary, but not sufficient, for efficient antitermination. In this study, we used the *Clostridium acetobutylicum alaS* gene, which encodes alanyl-tRNA synthetase, to investigate the specificity of the tRNA response. We show that the homologous *C. acetobutylicum* tRNA^Ala^ directs antitermination of the *C. acetobutylicum alaS* gene *in vitro*, but the heterologous *Bacillus subtilis* tRNA^Ala^ (with the same anticodon and acceptor end) does not. Base substitutions at positions that vary between these two tRNAs revealed synergistic and antagonistic effects. Variation occurs primarily at positions that are not conserved in tRNA^Ala^ species, which indicates that these non-conserved residues contribute to optimal antitermination of the homologous *alaS* gene. This study suggests that elements in tRNA^Ala^ may have coevolved with the homologous *alaS* T box leader RNA for efficient antitermination.

## 1. Introduction

Gram-positive bacteria utilize the T box riboswitch to regulate the expression of genes that encode aminoacyl-tRNA synthetases, amino acid biosynthesis enzymes, and transporters [1,2]. The leader region of most genes in the T box family contains an intrinsic transcriptional terminator that prevents transcription of the downstream gene; folding of the nascent transcript into a competing antiterminator structure, which is stabilized by binding of the cognate uncharged tRNA, allows synthesis of the full-length transcript. The anticodon of either charged or uncharged cognate tRNA pairs with a 3 nt sequence (designated the Specifier Sequence) that represents a codon that matches the amino acid identity of the downstream gene [3]. Stabilization of the antiterminator requires a second interaction between the free acceptor end (NCCA) of the uncharged tRNA and a complementary sequence (UGGN) embedded in the antiterminator bulge [4]; this interaction is prevented by the amino acid at the 3’ end of charged tRNA. A similar set of interactions regulates a subset of genes in the T box family at the level of translation initiation [5]. Therefore, expression of the downstream genes is induced when aminoacylation of the cognate tRNA is reduced.

Most T box RNAs contain a set of the canonical structural domains that define the T box riboswitch, including Stems I, II, and III, the IIA/B pseudoknot, and mutually exclusive terminator/antiterminator structures [2,6]. The *glyQS* leader RNA is a member of a rare class of T box RNAs that lack the Stem II and IIA/B pseudoknot domains [7]. tRNA^Gly^-dependent antitermination of the *glyQS* gene was demonstrated *in vivo* and *in vitro* [7], and this system was used in a variety of biochemical studies to investigate the requirements for efficient antitermination [8,9,10,11,12]. To determine if the features uncovered with *glyQS* are unique to that class or are generalizable to other genes in the T box family, we investigated another T box RNA with a similar deletion of Stems II and IIA/B that responds to a different tRNA.

**Figure 1:**
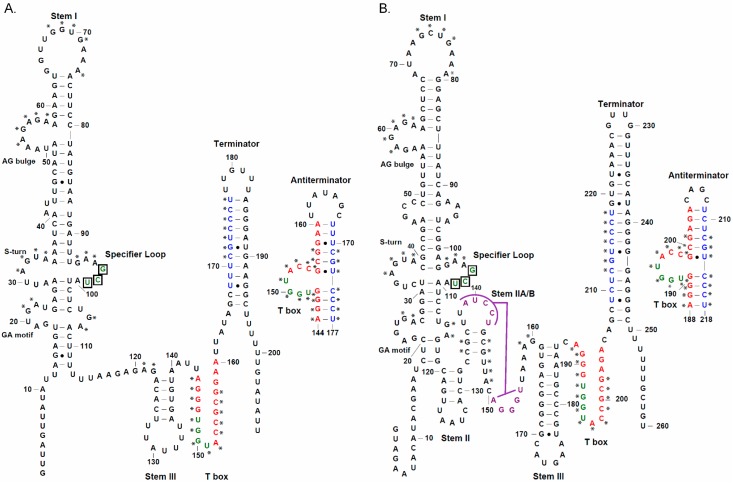
The predicted structural models of *alaS* T box leader RNAs. (**A**) *C. acetobutylicum alaS.* (**B**) *B. subtilis alaS*. Numbers start at the predicted transcription start-site (+1) for each gene. The RNAs are shown in the terminator form, and the alternative antiterminator structures are shown to the right of the terminator structures. Conserved sequence and structural elements, including the GA motif, S-turn, AG bulge, Specifier Loop and T box, and the pseudoknot (purple) are labeled. Asterisks indicate highly conserved nucleotides. Bases on the 5’ side of the terminator (blue) pair with part of the conserved T box sequence (red) to form the antiterminator. The GCU alanine Specifier Sequence (boxed) that pairs with the anticodon of tRNA^Ala^, and the UGGU residues in the antiterminator bulge that pair with the ACCA acceptor end of tRNA^Ala^, are shown in green. Watson-Crick pairs are indicated by “-”, wobble pairs are indicated by “•”.

Unlike glycyl genes, all of which lack Stems II and IIA/B, *alaS* genes (which encode alanyl-tRNA synthetase) include both deletion variants and members of the canonical class. In this study, we compared the *Clostridium acetobutylicum* and *Bacillus subtilis alaS* leader RNAs (Figure 1). The *C. acetobutylicum alaS* leader RNA, like the *glyQS* leader RNA, lacks the Stem II and IIA/B elements; other structural domains, including Stem I, III and the terminator and antiterminator, match the canonical pattern. In contrast, the *B. subtilis alaS* leader RNA, like most T box leader RNAs, includes the Stem II and IIA/B elements; however, the conserved Loop E motif in Stem II [6], which is predicted to form an S-turn, is missing. This motif, which is composed of opposing 5’-AGUA-3’ and 5’-GAA-3’ residues in an internal loop, is found in both the Specifier Loop and the Stem II domains of most T box RNAs.

The *B. subtilis alaS* leader RNA also has a noncanonical GA motif below the Specifier Loop. The canonical GA motif is composed of two short helices connected by a 3-nt bulge; there are two noncanonical pairs (G•A and A•G) in one helix, and three canonical W-C pairs in the other. This motif forms a kink-turn structure and is found at the base of Stem I in most T box leader RNAs [13]. The GA motif in the *B. subtilis alaS* leader RNA contains a 4-nt bulge, and the helix below the bulge has a U•U mismatch that separates the two C-G pairs. Noncanonical kink-turn elements have been reported previously [13,14,15], with variations in the bulge region or the pairing interaction in the helices. Therefore, it is possible that the *B. subtilis alaS* leader RNA forms a kink-turn, but the structural arrangement may be different from the canonical element found in other T box leader RNAs.

**Figure 2:**
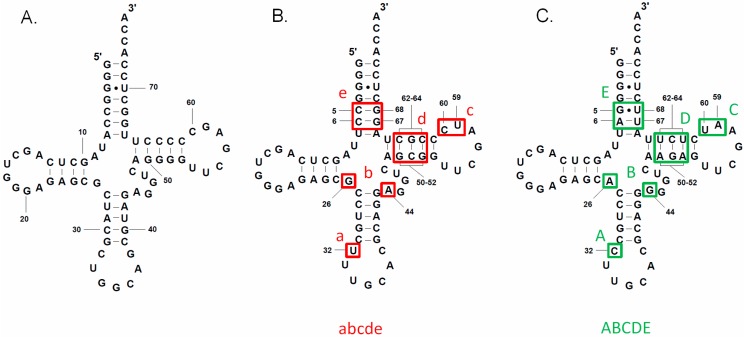
Cloverleaf diagrams of tRNA^Ala^. (**A**) *B. subtilis* tRNA^Ala^ (GGC). (**B**) *B. subtilis* tRNA^Ala^ (UGC). (**C**) *C. acetobutylicum* tRNA^Ala^ (UGC). Sequence differences between the heterologous tRNA^Ala^ (UGC) molecules ((B) and (C)) are boxed and assigned a letter (A/a, B/b, C/c, D/d and E/e); lower case letters in red represent sequences found in *B. subtilis* tRNA^Ala^ (UGC) (abcde), and upper case letters in green represent sequences found in *C. acetobutylicum* tRNA^Ala^ (UGC) (ABCDE). tRNA numbers are assigned based on tRNA numbering rules [23].

The Specifier Sequence of both *alaS* leader RNAs is a GCU alanine codon, which lacks the C residue at position 3 of the Specifier Sequence found in most T box leader RNAs [1]. The presence of the same Specifier Sequence suggested that both genes should respond to the same tRNA ligand. Two tRNA^Ala^ isoacceptors, with GGC or UGC anticodons, are encoded in the *B. subtilis* genome (Figure 2A,B). *B. subtilis* tRNA^Ala^ (UGC) has a 5-methoxyuridine (mo^5^U) at position 1 of the anticodon, while tRNA^Ala^ (GGC) has no anticodon modification; both tRNA^Ala^ isoacceptors are predicted to recognize a GCU alanine codon [16,17], and both contain an A at the discriminator position (residue 73, immediately preceding the 3’ CCA) that is complementary to the U at the variable position of the *alaS* antiterminator bulge; therefore, either could be a potential effector for *alaS* antitermination. In contrast, the *C. acetobutylicum* genome contains only one tRNA^Ala^ isoacceptor, which has a UGC anticodon and A at the discriminator position (Figure 2C); this tRNA^Ala^ (UGC) is therefore predicted to be the effector molecule for the *alaS* gene in this organism, despite a U•U mismatch at the third position of the Specifier Sequence. *B. subtilis* tRNA^Ala^ (UGC) and *C. acetobutylicum* tRNA^Ala^ (UGC), which have the same anticodon and acceptor end, have sequence variations at several regions (Figure 2B,C). Two of these, at the anticodon loop (position 32) and central core (positions 26 and 44), show anticodon-dependent sequence conservation in many bacterial tRNA species [18]. Mutations in these regions in tRNA^Gly^ affect *glyQS* antitermination [19]. However, the residue at position 32 in tRNA^Ala^ (UGC) is less conserved than in tRNA^Gly^ (GCC), and residues at positions 26 and 44 are not conserved in either tRNA^Ala^ (UGC) or tRNA^Gly^ (GCC) [18]. Therefore, it is unclear whether tRNA residues at these positions play a role in *alaS* antitermination similar to that observed for *glyQS*.

Although correct Specifier Sequence-anticodon and acceptor end-antiterminator bulge pairings are necessary for antitermination, tRNA elements outside of these two major sites also contribute to efficient interaction with the cognate leader RNA [8,19,20]. For *glyQS*, this includes an interaction between the Stem I terminal platform and the elbow domain of tRNA^Gly^ [21,22]. However, the residues that participate in this interaction in *glyQS* are unique to glycyl genes, suggesting that this interaction may operate differently in other classes of T box genes. This study investigates tRNA determinants important for efficient antitermination of the *alaS* gene, in comparison to previous findings for the *glyQS* and *tyrS* genes, to identify general features of tRNA recognition by T box leader RNAs of different structural classes. tRNA recognition by the translational machinery places numerous constraints on tRNA sequence and structure. This work provides insight into how tRNA sequence requirements for antitermination are superimposed upon translational requirements.

## 2. Experimental Section

### 2.1. Generation of DNA Templates

A *C. acetobutylicum alaS*-*lacZ* transcriptional fusion, which has the *C. acetobutylicum alaS* promoter region and leader sequence fused to a *lacZ* reporter gene in plasmid pFG328 [24], was used to generate PCR templates for *in vitro* transcription. The resulting 374 bp DNA fragment extends from 119 bp upstream of the transcription start-site (+1) to 53 bp downstream of the predicted termination site (+202). A similar *B. subtilis alaS*-*lacZ* transcriptional fusion was constructed in plasmid pFG328 and used to generate a 510 bp template fragment that extends from 120 bp upstream of the +1 site to 134 bp downstream of the predicted termination site (+265).

Templates for transcription of tRNA by T7 RNA polymerase (RNAP) contained the T7 RNAP promoter sequence positioned at position 1 of the tRNA, and were constructed by oligonucleotide cassette ligation and amplified by PCR as described previously [8]. PCR templates were purified using a QIAquick PCR purification kit (Qiagen, Valencia, CA USA) and confirmed by sequencing (Genewiz, Inc., South Plainfield, NJ, USA).

### 2.2. T7 RNAP Transcription

T7 RNAP transcription of tRNA was carried out by overnight incubation of DNA templates in 40 mM Tris-HCl [pH 8], 2 mM spermidine, 22 mM MgCl_2_, 5 mM DTT, 4 mM NTP, 4 mM GMP, 0.028 μM pyrophosphatase (Roche, Indianapolis, IN USA), 8 U RNase inhibitor (Roche) and 0.45 μM T7 RNAP (Ambion) at 37 °C. RNA products were purified on a 6% (*w/v*) denaturing polyacrylamide gel, and quantified with an ND1000 Spectrophotometer (NanoDrop Technologies). The resulting tRNAs were folded in the presence of 10 mM MgCl_2_ by heating to 80 °C for 2 min followed by slow cooling over 40 min to room temperature.

### 2.3. In Vitro Transcription Termination Assays

Single round transcription reactions were carried out as described previously [7] with some modifications. Transcription reactions were initiated in the presence of 1 nM PCR template, 140 μM GpU dinucleotide (Sigma), 6 nM His-tagged purified *B. subtilis* RNAP, 2.5 μM ATP and GTP, 0.75 μM UTP and 0.25 μM [α-^32^P] UTP (800 Ci/mmol; 1 Ci = 37 GBq) in 1X transcription buffer (20 mM Tris-HCl [pH 7.9], 10 mM MgCl_2_, 20 mM NaCl, 0.1 mM EDTA) at 37 °C for 15 min. Omission of CTP resulted in a halt at position +39 of the *C. acetobutylicum alaS* leader template, and at position +9 of the *B. subtilis alaS* template. Heparin (Sigma, St. Louis, MO USA) was added to the halted complexes at 25 nM to prevent reinitiation, and transcription elongation was restarted by addition of NTPs (10 μM final concentration) in the presence of increasing amounts of tRNA^Ala^ (final concentration 0, 0.25, 0.5, 1, 5, 10 μM). After 15 min incubation at 37 °C, reactions were stopped by addition of 3X stop buffer (7 M urea, 0.1 M EDTA, 4.8% glycerol, xylene cyanol and bromophenol blue) and resolved on 6% denaturing gels. The results were analyzed on a PhosphorImager (Amersham Biosciences, Piscataway, NJ, USA) and quantified by ImageQuant 5.2 software. The percent readthrough (% RT) was calculated by dividing the amount of readthrough transcript by the total transcript (termination and readthrough products) and multiplying by 100. The % RT determined in the absence of tRNA was subtracted from each result to eliminate tRNA-independent readthrough, and the resulting values (corrected % RT) were fit to a hyperbolic equation to calculate the *K*_1/2_ and RT_max_ as described previously [19].

### 2.4. Genetic Techniques

The *B. subtilis alaS-lacZ* transcriptional fusion construct was introduced into *B. subtilis* strain ZB307A (SPβ*c2del2*::Tn*917*::pSK10Δ6) by transformation to allow integration into the SPβ prophage through homologous recombination, and transformants were selected using medium containing 5 µg/mL chloramphenicol [25]. Phage carrying the *alaS*-*lacZ* fusion were purified by passage through *B. subtilis* strain ZB449 (*trpC2 pheA1 abrB703*, SPβ cured) [26] and introduced into *B. subtilis* strain 1A434 (*ala-1 leuB8 metA5 pur thrC5 trpC*) from the *Bacillus* Genetic Stock Center (Ohio State University) by transduction.

### 2.5. Bacterial Growth Conditions and β-galactosidase Assays

For alanine starvation experiments, *B. subtilis* strain 1A434 carrying an *alaS*-*lacZ* transcriptional fusion was grown to mid-exponential phase in minimal medium [27] with 5 µg/mL chloramphenicol and 50 µg/mL of the appropriate amino acids (l-alanine, l-leucine, l-methionine, l-threonine, l-tryptophan) and adenine, harvested and resuspended in fresh medium with or without 50 µg/mL L-alanine. Cells were collected at 1 h intervals for β-galactosidase assays.

## 3. Results

### 3.1. tRNA^Ala^-Directed alaS Antitermination in Vitro

**Figure 3:**
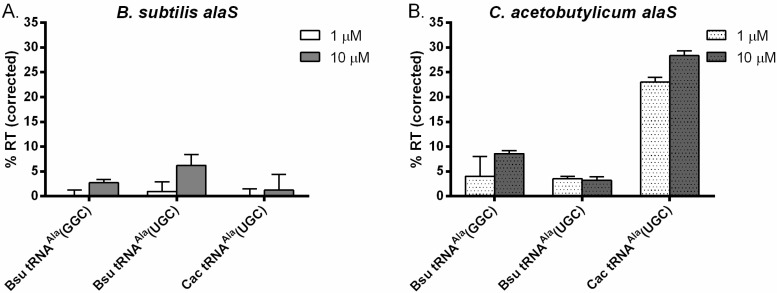
Antitermination of *alaS* genes *in vitro*. (**A**) *B. subtilis alaS.* (**B**) *C. acetobutylicum alaS.* Transcription was carried out in the presence of 1 or 10 µM T7 RNAP-transcribed tRNA^Ala^ and percent readthrough (% RT) was corrected for tRNA-independent readthrough. All experiments were repeated at least twice and error bars represent the standard error of the mean (SEM). *Bsu*, *B. subtilis*; *Cac*, *C. acetobutylicum.*

We tested the ability of *B. subtilis* and *C. acetobutylicum* tRNA^Ala^ isoacceptors to mediate antitermination of both the *B. subtilis* and *C. acetobutylicum alaS* genes *in vitro*. Antitermination of the *glyQS* gene reaches saturation at 0.5 µM tRNA^Gly^ [19]. Therefore, we initially tested tRNA^Ala^ at 1 and 10 µM. For the *B. subtilis alaS* gene, no antitermination was observed using either of the *B. subtilis* tRNA^Ala^ isoacceptors or *C. acetobutylicum* tRNA^Ala^ (UGC) at 1 µM (Figure 3A). At 10 µM, *B. subtilis* tRNA^Ala^ (GGC) and tRNA^Ala^ (UGC) conferred readthrough at 4% and 8%, respectively (Figure 3A), indicating that *B. subtilis* tRNA^Ala^ (UGC) is somewhat more effective than tRNA^Ala^ (GGC), despite the fact that the UGC anticodon forms a less stable U•U mismatch at position 3 of the GCU Specifier Sequence than the U•G wobble pair formed by the GGC anticodon. *C. acetobutylicum* tRNA^Ala^ (UGC) resulted in poor (~2%) antitermination of the *B. subtilis alaS* gene. It appears that antitermination of the *B. subtilis alaS* gene is not very efficient under these conditions with any of the tRNA^Ala^ variants tested. This is consistent with the general observation that leader RNAs with the complete canonical structure (including Stems II and IIA/B) exhibit low antitermination efficiency *in vitro* [7,28].

In contrast to the *B. subtilis alaS* gene, the *C. acetobutylicum alaS* gene exhibited more efficient antitermination by the cognate *C. acetobutylicum* tRNA^Ala^ (UGC) (23% at 1 µM, 28% at 10 µM; Figure 3B). This indicates that *in vitro* antitermination by the homologous tRNA is more efficient for the *C. acetobutylicum alaS* leader RNA, which lacks Stems II and IIA/B, than for the canonical *B. subtilis alaS* gene. *B. subtilis* tRNA^Ala^ (GGC) at 10 µM resulted in ~10% readthrough of the *C. acetobutylicum alaS* terminator; this activity was two-fold greater than that observed for *B. subtilis* tRNA^Ala^ (UGC) (Figure 3B). This is opposite to what was observed for the *B. subtilis alaS* gene, where *B. subtilis* tRNA^Ala^ (UGC) was two-fold more efficient than tRNA^Ala^ (GGC), indicating that the tRNA requirements for the *C. acetobutylicum alaS* gene are different from those for the *B. subtilis alaS* gene. Although the GCU•GGC Specifier Sequence-anticodon pairing by *B. subtilis* tRNA^Ala^ (UGC) results in a U•G wobble pair at position 3 of the Specifier Sequence, which may allow more efficient interaction than the U•U mismatch formed by tRNA^Ala^ (UGC), the homologous *C. acetobutylicum* tRNA^Ala^ (UGC) also forms a U•U mismatch at position 3. This suggests that the U•U mismatch is not the basis for poor antitermination by *B. subtilis* tRNA^Ala^ (UGC), and that there are requirements for efficient antitermination in addition to the pairing stability between the Specifier Sequence and anticodon.

**Figure 4:**
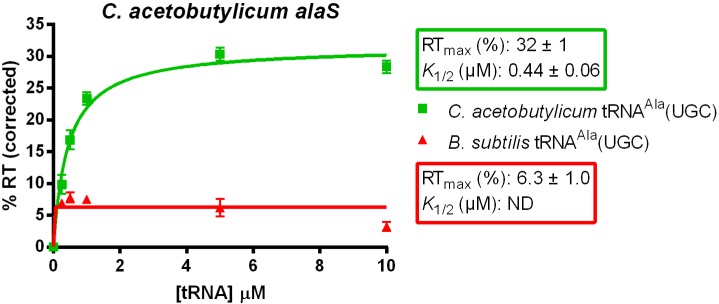
Antitermination of the *C. acetobutylicum alaS* gene using *C. acetobutylicum* and *B. subtilis* tRNA^Ala^ (UGC). Single round transcription reactions of the *C. acetobutylicum alaS* leader template were performed in the presence of increasing amounts of tRNA^Ala^ (final concentration 0, 0.25, 0.5, 1.0, 5.0, 10 μM). tRNA-independent readthrough was subtracted from each data point, and the resulting values were fit to a hyperbolic equation to determine the *K*_1/2_ and RT_max_. All experiments were repeated at least twice and error bars represent the standard error of the mean (SEM). ND (not determined) indicates that the *K*_1/2_ value was too high to be determined.

We compared the antitermination efficiency (*K*_1/2_, the tRNA concentration required to reach 50% of the maximum readthrough, RT_max_) of the *C. acetobutylicum alaS* gene using either homologous or heterologous tRNA^Ala^ (UGC) (Figure 4) to allow quantitative comparison between the two tRNA constructs [19]. The RT_max_ indicates the functional leader RNA-tRNA interaction at saturating tRNA, while the *K*_1/2_ represents the efficiency of the interaction between the two molecules. The homologous *C. acetobutylicum* tRNA^Ala^ directed antitermination of the *C. acetobutylicum alaS* leader RNA with a *K*_1/2_ = 0.44 µM; antitermination by the heterologous *B. subtilis* tRNA^Ala^ (UGC) was too low (5% RT_max_ at saturating tRNA) to determine the *K*_1/2_, despite 80% sequence identity between the two tRNAs. For comparison, tRNA^Gly^-dependent antitermination of *B. subtilis glyQS* gives a *K*_1/2_ = 0.063 µM and an RT_max_ of 94% [19]. These variations in activity could be due to different experimental requirements for optimal antitermination of the *alaS* and *glyQS* genes *in vitro*, or different inherent requirements for expression of the *alaS* and *glyQS* genes in each organism. The failure of *B. subtilis* tRNA^Ala^ (UGC) to direct efficient antitermination of the *C. acetobutylicum alaS* gene suggests a requirement for tRNA elements outside of the known anticodon and acceptor end interaction positions, as the two tRNAs are identical at those positions.

### 3.2. tRNA^Ala^ Requirements for alaS Antitermination

We compared the sequences of *C. acetobutylicum* tRNA^Ala^ (UGC) and *B. subtilis* tRNA^Ala^ (UGC) to identify elements that may contribute to the differences in antitermination efficiency. Variations at five regions, including positions 32 (anticodon loop), 26 and 44 (central core), 59 and 60 (T loop), and pairings at 50-64, 51-63, 52-62 (T stem), and 5-68, 6-67 (acceptor stem), were identified (Figure 2B,C). Only position 32 and the 52-62 pair in the T stem showed moderate anticodon-dependent conservation among tRNA^Ala^ (UGC) species [18]; the variable residues at other positions are not conserved in tRNA^Ala^ (UGC). Upper-case (*C. acetobutylicum*) and lower-case (*B. subtilis*) letters were assigned to represent the nucleotides at each position.

**Figure 5:**
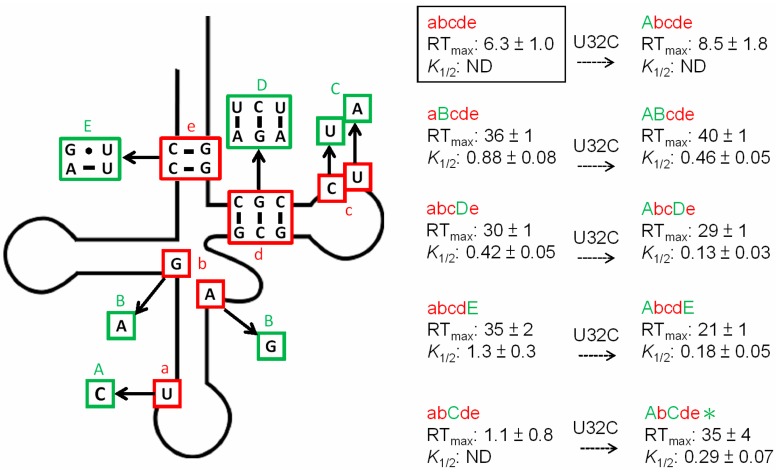
Effects of tRNA mutations on antitermination. Cloverleaf model of *B. subtilis* tRNA^Ala^ (UGC) with sequences shown at five regions (abcde, red) where there are differences from *C. acetobutylicum* tRNA^Ala^. The corresponding sequences from *C. acetobutylicum* tRNA^Ala^ (UGC) (ABCDE, green) were introduced as indicated. The resulting constructs were tested for antitermination of the *C. acetobutylicum alaS* template to determine the RT_max_ and *K*_1/2_. Asterisk (*) indicates that the RT_max_ and *K*_1/2_ were calculated without the values determined at the highest two tRNA concentrations. All experiments were repeated at least twice and error bars represent the standard error of the mean (SEM). ND (not determined) indicates that the *K*_1/2_ value was too high to be determined accurately.

Elements “abcde” found in *B. subtilis* tRNA^Ala^ (UGC) were replaced individually by elements “ABCDE” found in *C. acetobutylicum* tRNA^Ala^ (UGC) to identify substitutions that promote efficient *C. acetobutylicum alaS* antitermination. A U32C anticodon loop mutation (a➔A) in *B. subtilis* tRNA^Ala^ (UGC) had no effect on antitermination of the *C. acetobutylicum alaS* gene (Abcde, Figure 5). A U32C substitution in tRNA^Gly^ enhanced antitermination of a *glyQS* construct with a GGU•UCC Specifier Sequence-anticodon pair, which also has a U•U mismatch at position 3 of the Specifier Sequence [19]; the lack of effect of a U32C mutation in tRNA^Ala^ (UGC) indicates differences in the role of this nucleotide in different tRNA contexts. The tRNA^Ala^ variant with G26A•A44G substitutions (b➔B, aBcde) exhibited a substantial increase in antitermination efficiency, but was two-fold less efficient than *C. acetobutylicum* tRNA^Ala^ (UGC) (*K*_1/2_ 0.88 *vs.* 0.44 for *C. acetobutylicum* tRNA^Ala^). The addition of U32C (ABcde) resulted in an increase in antitermination efficiency to a level similar to that of the *C. acetobutylicum* tRNA (Figure 5). This indicates that base substitutions at the central core contribute to efficient *C. acetobutylicum alaS* antitermination, which is enhanced by the anticodon loop substitution, despite the fact that the U32C substitution alone had no effect. This result indicates that U32C can enhance antitermination activity, as was observed in *glyQS* antitermination, but the effect is dependent on other tRNA features.

A *B. subtilis* tRNA^Ala^ (UGC) variant with nucleotides transplanted from the T stem of *C. acetobutylicum* tRNA^Ala^ (UGC) (G50A-C64U, C51G-G63C, G52A-C62U, d➔D, abcDe) directed antitermination with an efficiency similar to that of *C. acetobutylicum* tRNA^Ala^ (UGC), and introduction of U32C (AbcDe) resulted in a three-fold increase in antitermination relative to the construct with T stem substitutions alone (Figure 5). The T stem substitutions are predicted to reduce the stability of the helix, which suggests that destabilization of the T stem of *B. subtilis* tRNA^Ala^ (UGC) is sufficient for efficient *alaS* antitermination.

A *B. subtilis* tRNA^Ala^ (UGC) variant with acceptor stem substitutions (C5G-G68U, C6A-G67U, e➔E, abcdE) resulted in an RT_max_ similar to that of *C. acetobutylicum* tRNA^Ala^ (UGC), but a three-fold increase in *K*_1/2_, indicating a less efficient interaction. These substitutions are predicted to reduce the pairing stability at the acceptor stem, similar to the effect of the T stem substitutions. In agreement with what was observed for the T stem substitutions, introduction of the U32C mutation into the tRNA variant with acceptor stem substitutions resulted in a seven-fold increase in efficiency (lower *K*_1/2_); however, this was accompanied by a reduction in the RT_max_ from 35% to 21% (AbcdE). A similar effect was observed when a U32C mutation was introduced into tRNA^Gly^ in the context of the wild-type *glyQS* leader RNA-tRNA^Gly^ (GCC) interaction [19]. This result indicates that the anticodon loop and acceptor stem substitutions in tRNA^Ala^ may affect *alaS* antitermination similarly to *glyQS*.

Substitutions at the T loop (U59A/C60U, c➔C, abCde) of *B. subtilis* tRNA^Ala^ (UGC) completely abolished antitermination (abCde, Figure 5). However, the introduction of U32C into this tRNA construct (AbCde) resulted in antitermination similar to that of *C. acetobutylicum* tRNA^Ala^ (UGC). Because neither the T loop nor the anticodon loop substitutions in *B. subtilis* tRNA^Ala^ (UGC) promoted antitermination, the observation that both substitutions in combination enhanced antitermination indicates a synergistic effect.

In sum, we found that base substitutions in *B. subtilis* tRNA^Ala^ (UGC) at the central core, the T stem or the acceptor stem with nucleotides found in *C. acetobutylicum* tRNA^Ala^ (UGC) enhanced *C. acetobutylicum alaS* antitermination. This suggests that residues at these positions are not directly recognized by the *alaS* leader RNA. Instead, they may have structural effects on tRNA^Ala^ (e.g., destabilization of pairing interactions or organization of the anticodon loop) that allow efficient *alaS* antitermination. In some cases (e.g., U32C and U59A/C60U), combination of the mutations had a synergistic effect, while other combinations resulted in decreased antitermination.

### 3.3. Non-Conserved Residues Contribute to tRNA^Ala^ Tuning for alaS Antitermination

**Figure 6:**
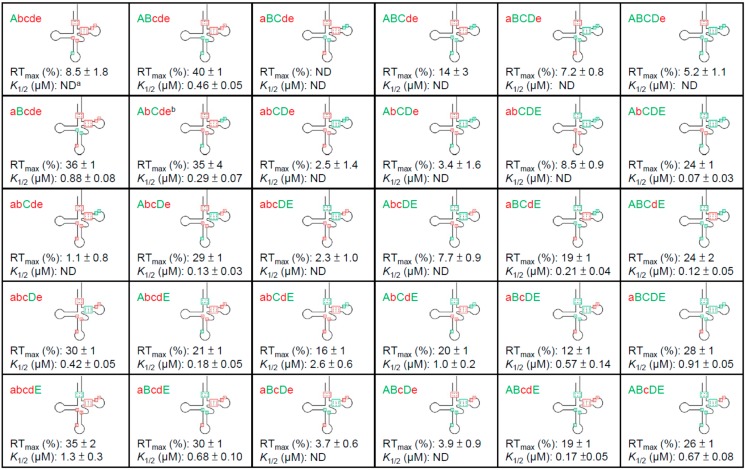
Antitermination of *C. acetobutylicum alaS* leader RNA using tRNA^Ala^ mutants. Elements ABCDE found in *C. acetobutylicum* tRNA^Ala^ (UGC) were sequentially introduced into *B. subtilis* tRNA^Ala^ (UGC), and the resulting constructs were tested for *C. acetobutylicum alaS* antitermination to determine RT_max_ and *K*_1/2_. ^a^: ND not determined. ^b^: The *K*_1/2_ and RT_max_ were calculated without the values of 0.2 and 0.5 µM tRNA.

We tested all possible combinations of base substitutions at the five positions that differ between *B. subtilis* and *C. acetobutylicum* tRNA^Ala^ (UGC), and list the effects of each tRNA construct on *C. acetobutylicum alaS* antitermination (Figure 6). Thirteen of the 30 constructs showed *K*_1/2_ over the detection limit of the *in vitro* transcription assays (>160 µM), indicating poor activity. 10 of these 13 variants contain the stable C5-G68 and C6-G67 pairs (element “e”), which suggests that stable pairings at the acceptor arm of tRNA^Ala^ are detrimental to *C. acetobutylicum alaS* antitermination.

Five constructs (aBcde, abcDe, ABcde, AbcDe, AbCde, Figure 6) contain the stable acceptor end pairs but still directed functional antitermination. These constructs had changes at the central core (b➔B), the T stem (d➔D), or the anticodon loop (a➔A) in addition to the stable acceptor end. This suggests that residues at other positions of tRNA can offset the detrimental effect of the stable acceptor end pairs.

Three constructs (abcDE, abCDE and AbcDE, Figure 6) that contain the less stable G5-U68 and A6-U67 pairs (E) also showed *K*_1/2_ values over the detection limit (ND); this is opposite to what we expected because these variants do not have the stable element e that is detrimental to antitermination. The common feature of these constructs is that in addition to element E, they also contain G26•A44 (b), and A50-U64, G51-C63 and A52-U62 (D) pairs, indicating that the combination of these elements (b, D and E) resulted in detrimental effects similar to that of element e alone. The remaining 12 tRNA variants that have element E all conferred detectable antitermination efficiency (Figure 6), which indicates that the G5-U68 and A6-U67 pairs are important. These constructs resulted in a range of RT_max_ (12%–35%) and *K*_1/2_ (0.07–1.3 µM) values depending on the composition of the tRNA. The presence of U32 (a) generally decreased the RT_max_ and increased the *K*_1/2_, indicating reduced activity in most of the tRNA constructs. The exceptions included ABcdE *vs.* aBcdE, which resulted in increased RT_max_ (by 1.6-fold) and increased *K*_1/2_ (by 4.0-fold), indicating lower efficiency at low tRNA concentration, and ABcDE *vs.* aBcDE, which resulted in similar *K*_1/2_ but a two-fold decrease in RT_max_. These results indicate that the residue at position 32 causes different effects depending on other tRNA elements.

Substitutions at the central core (B➔b), T loop (C➔c) or T stem (D➔d) also had different effects depending on the context (Figure 6). For example, introduction of central core substitutions (G26A•A44G, B➔b) into *C. acetobutylicum* tRNA^Ala^ (UGC) (AbCDE) resulted in reduced RT_max_ (from 32% to 24%) and *K*_1/2_ (by 6.3-fold); this indicates that the maximum antitermination at saturating tRNA is reduced, but the efficiency at low tRNA concentration greatly increased. Similar results were observed for T stem substitutions (A50G-U64C, G51C-C63G, A52G-U62C, D➔d) in *C. acetobutylicum* tRNA^Ala^ (UGC) (ABCdE). However, when both substitutions were introduced (AbCdE), the RT_max_ was reduced from 32% to 20% while the *K*_1/2_ increased by 8.6-fold in comparison with *C. acetobutylicum* tRNA^Ala^ (UGC); this indicates that substitutions at the central core and T stem antagonized each other and resulted in reduced efficiency, despite the observation that each substitution alone had a positive effect.

We also observed that introduction of the A59U/U60C substitutions (C ➔c) into *C. acetobutylicum* tRNA^Ala^ (UGC) (ABcDE) reduced RT_max_ and increased *K*_1/2_, whereas the abCdE➔abcdE comparison, which contains the same C➔c substitution, resulted in increased RT_max_ and decreased *K*_1/2_ (Figure 6). This indicates that the same T loop substitutions in different tRNA contexts resulted in opposite effects on antitermination.

**Figure 7:**
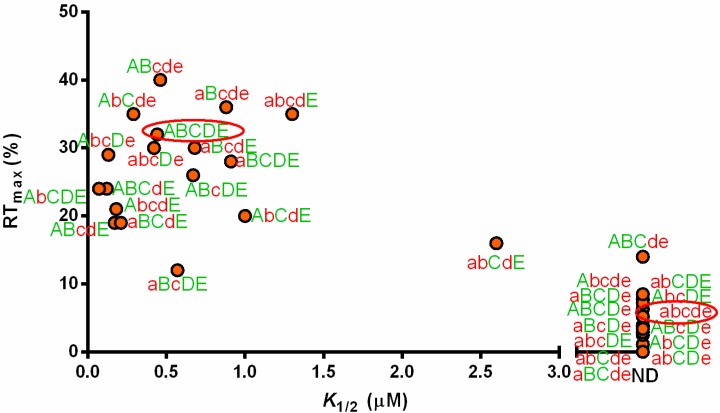
Antitermination of *C. acetobutylicum alaS* by tRNA^Ala^ (UGC). The RT_max_ and *K*_1/2_ for each construct were determined by *in vitro* antitermination assays. *C. acetobutylicum* tRNA^Ala^ (ABCDE) and *B. subtilis* tRNA^Ala^ (abcde) are circled. ND (not determined) indicates that the *K*_1/2_ value was too high to be determined accurately.

The relationship between RT_max_ and *K*_1/2_ of all tRNA constructs tested is shown in Figure 7. As noted above, the same substitutions often had different effects in different tRNA contexts, resulting in different effects on antitermination. This suggests that the elements at these five positions are not directly recognized by the leader RNA, but instead collaborate to tune the tRNA structure to achieve efficient antitermination.

### 3.4. Expression of alaS-lacZ Fusions in B. subtilis

Antitermination of the *B. subtilis alaS* gene by either of the *B. subtilis* tRNA^Ala^ isoacceptors was not efficient *in vitro*; this may be due to the structural complexity of the *B. subtilis alaS* leader RNA or may reflect sensitivity to the absence of tRNA modification. Therefore, we generated *lacZ* reporter fusions to test the ability of modified tRNA^Ala^ to mediate antitermination of the *C. acetobutylicum* and *B. subtilis alaS* gene *in vivo* under alanine starvation conditions. Expression of the *B. subtilis alaS*-*lacZ* gene increased two-fold when cells were starved for alanine (Table 1), which indicates that the *B. subtilis alaS* T box leader RNA responds to an increase in uncharged tRNA^Ala^, albeit weakly. In contrast, no significant induction of the *C. acetobutylicum alaS*-*lacZ* fusion was observed. This suggests that *B. subtilis* tRNA^Ala^ can direct antitermination of the *B. subtilis alaS* gene, but not the *C. acetobutylicum alaS* gene; the failure of the heterologous *B. subtilis* tRNA to mediate antitermination of the *C. acetobutylicum alaS* gene is consistent with the poor antitermination observed *in vitro* using *B. subtilis* tRNA^Ala^.

**Table 1:** Expression of *alaS*-*lacZ* fusions in *B. subtilis*.

	β-galactosidase activity (Miller units) ^a^		induction ratio
	uninduced	induced	
*B. subtilis alaS*-*lacZ*	11 ± 1^b^	22 ± 1	2.0
*C. acetobutylicum alaS*-*lacZ*	5.9 ± 1.7	6.4 ± 1.3	1.1

^a^ Fusions were integrated in single copy into an alanine auxotroph, and cells were grown in the presence or absence of alanine to induce accumulation of uncharged tRNA^Ala^. ^b^ Results are shown as mean ± SEM.

## 4. Discussion

Previous studies on antitermination of the *glyQS* and *tyrS* genes identified tRNA requirements for efficient antitermination [8,20,29]. However, the results were hard to compare due to differences between *in vitro* and *in vivo* experimental conditions, and to the use of genes in different aaRS families. The requirements for antitermination of leader RNAs that lack Stems II and IIA/B may differ from those with the canonical structure. In this study, we established an *in vitro* system for the *C. acetobutylicum alaS* gene and investigated tRNA^Ala^ requirements for efficient *alaS* antitermination.

We observed that *in vitro* antitermination of the *B. subtilis alaS* leader RNA (with the canonical structural arrangement) is much less efficient than antitermination of the *C. acetobutylicum alaS* leader RNA (without Stems II and IIA/B). This result is consistent with our previous analyses of the *tyrS* and *glyQS* genes, where tRNA-dependent antitermination could be demonstrated using the *glyQS* natural deletion variant, but not the canonical *tyrS* leader RNA [7,30]. tRNA-dependent antitermination of leader RNAs with the Stem II and IIA/B elements was previously demonstrated *in vitro* using either cellular extracts or a high concentration of polyamine [28], suggesting that there are different requirements for antitermination of leader RNAs of different structural classes.

In this study, we showed that *C. acetobutylicum* tRNA^Ala^ (UGC) directs efficient *C. acetobutylicum alaS* antitermination *in vitro*, and less efficient *B. subtilis alaS* antitermination. In contrast, neither of the *B. subtilis* tRNA^Ala^ isoacceptors directed efficient antitermination of the *C. acetobutylicum alaS* gene *in vitro* and *in vivo*. A U•U mismatch at Specifier Sequence position 3 appears to be tolerated in this system, and efficiency may be enhanced by tRNA modification *in vivo*. Expression of a *B. subtilis alaS*-*lacZ* fusion was observed in *B. subtilis* cells under alanine starvation conditions, which indicates that *B. subtilis* tRNA^Ala^ can interact with the homologous *alaS* leader RNA *in vivo*. Together these results suggest that each T box leader RNA may have coevolved with the cognate tRNA in each organism to achieve interaction specificity.

**Figure 8:**
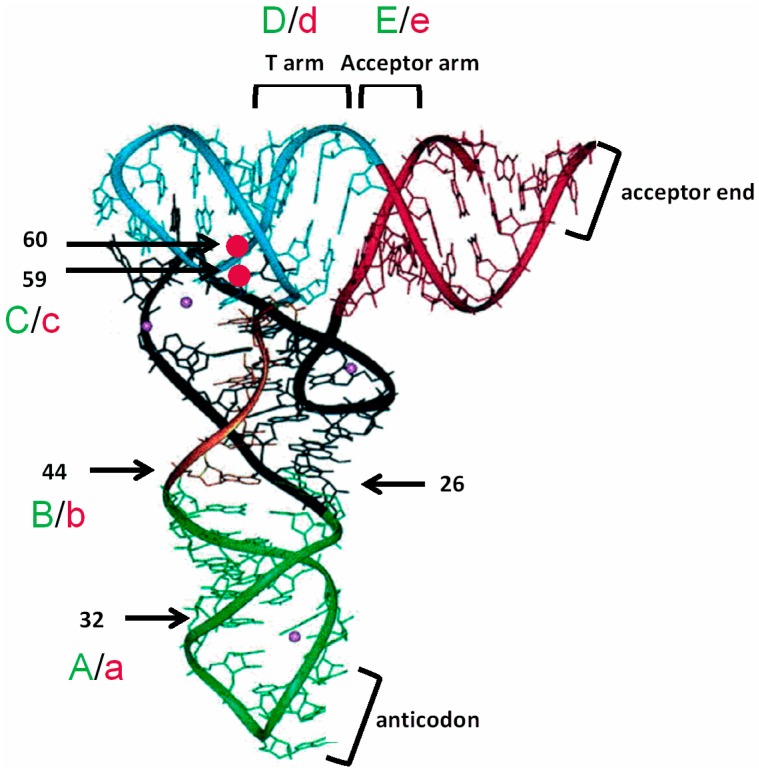
Positions of substitutions on tRNA tertiary structure. Yeast tRNA^Phe^ three-dimensional structure (adapted from [31]) is used to demonstrate the relative positions of elements tested in this study. Anticodon stem-loop (green), D stem-loop, (black), variable loop (orange), T stem-loop (blue), and acceptor arm (red). Locations of substitutions tested in this study are indicated.

Sequence comparison between *B. subtilis* tRNA^Ala^ (UGC) and C. *acetobutylicum* tRNA^Ala^ (UGC) revealed nucleotide variations at five regions (Figure 8). Substitutions in *B. subtilis* tRNA^Ala^ (UGC) at the central core, the T arm, or the acceptor arm with the corresponding residues from *C. acetobutylicum* tRNA^Ala^ (UGC) was sufficient to promote efficient *C. acetobutylicum alaS* antitermination *in vitro*. The observation that any of these substitutions is sufficient indicates that the residues at these positions are not specifically recognized by the leader RNA. Instead, the substitutions may tune the tRNA conformation to allow proper interaction with the *alaS* leader RNA, which implies that the leader RNA recognizes the overall tRNA structure rather than individual bases at these positions. This result is consistent with our previous *in vivo* and *in vitro* findings [11,20].

The U32C anticodon loop substitution was of particular interest. This mutation in tRNA^Gly^ was proposed to reorganize the anticodon loop conformation to promote an enhanced pairing interaction at the Specifier Sequence [19]. However, this substitution in *B. subtilis* tRNA^Ala^ (UGC) was not sufficient to allow efficient *C. acetobutylicum alaS* antitermination. The U32C mutation was effective only in combination with other substitutions, which suggests that there are additional requirements (e.g., proper presentation of the acceptor end of tRNA to the antiterminator bulge [19]) that are crucial for *alaS* antitermination. The presence of C38 in the tRNA^Ala^ anticodon loop (instead of A38 in tRNA^Gly^) also may result in a different anticodon loop geometry that influences the effect of the nucleotide at position 32.

The combination of U32C (a➔A) and the acceptor stem substitutions (e➔E) in *B. subtilis* tRNA^Ala^ (UGC) (AbcdE) resulted in reduced *K*_1/2_ and RT_max_ (indicating more efficient antitermination at low tRNA concentration but less efficient antitermination at saturating tRNA concentrations) as compared with the tRNA variant with only the acceptor stem substitutions (abcdE). A similar result was observed in *glyQS* antitermination when a U32C mutation was introduced into the wild-type *B. subtilis* tRNA^Gly^ (GCC) [19]. We hypothesize that U32C in tRNA^Gly^ results in enhanced interaction between the glycine Specifier Sequence and anticodon; however, a tightened interaction at the Specifier Sequence may constrain the positioning of the tRNA acceptor end relative to the antiterminator bulge, and therefore reduce the RT_max_. The results of the current study indicate that U32C also may enhance the interaction between the alanine Specifier Sequence and anticodon, and this effect may restrict the proper presentation of the acceptor end of the tRNA^Ala^ variant with acceptor stem substitutions to the antiterminator bulge.

We observed that the U59A/C60U mutations had positive or negative effects, depending on the tRNA context. The unpaired nucleotides 59 and 60 are located at the junction of the coaxially-stacked D/anticodon stem and T/acceptor stem helices in the tRNA L-shape structure (Figure 8). Substitions at positions 59 and 60 may alter the angle of the two stacking helices in tRNA, thereby affecting the interaction with the leader RNA. This could explain why U59A/C60U antagonized the positive effect of substitutions at the central core or T arm that resulted in efficient antitermination in the context of *B. subtilis* tRNA^Ala^ (UGC), but had a synergistic effect with U32C. Previous mutational analyses revealed that only certain combinations of bases at positions 59 and 60 were found in tRNA^Tyr^ constructs active in induction of *tyrS* expression *in vivo* [20], which indicates that the nucleotide identities at these positions are important for antitermination. The results of the current study show that base substitutions at positions 59 and 60 alter *alaS* antitermination efficiency, which is consistent with the findings in the *tyrS* system.

Substitutions at the central core, the T stem or the acceptor stem of *B. subtilis* tRNA^Ala^ (UGC) also may affect the presentation of the acceptor end. The X-ray crystal structure of the *glyQS* Stem I-tRNA^Gly^ complex revealed a distortion at positions 26 and 44 of tRNA^Gly^ [21]. The resulting tRNA structure mimics the bent structure of tRNA^Phe^ in the P/P state during translation. The G26A•A44G substitutions in *B. subtilis* tRNA^Ala^ (UGC) may reorient the conformation of the central core, leading to proper presentation of the acceptor end sequence for efficient antitermination. Because base pairs in the T arm and the acceptor arm of the *B. subtilis* tRNA^Ala^ (UGC) are more stable than those in *C. acetobutylicum* tRNA^Ala^ (UGC), substitutions d➔D and e➔E in *B. subtilis* tRNA^Ala^ (UGC) may increase the flexibility of these regions, which facilitates the interaction between the acceptor end and the antiterminator bulge.

The G3•U70 pair is a highly conserved, major tRNA^Ala^ recognition determinant for AlaRS [32,33]. A U70C mutation, which confers a more stable G-C pair, resulted in enhanced antitermination efficiency (RT_max_ = 48% ± 1; *K*_1/2_ = 0.33 ± 0.03 μM) relative to the wild-type *C. acetobutylicum* tRNA^Ala^ (RT_max_ = 32% ± 1; *K*_1/2_ = 0.44 ± 0.06 μM). In contrast, stabilization of the 5-68 and 6-67 pairs (e➔E) resulted in reduced antitermination. Because the acceptor stem adopts an A-form RNA conformation, which is composed of 11 bp per turn [8,34], stabilization of the pairings at 5-68 and 6-67 in *C. acetobutylicum* tRNA^Ala^ (UGC) may shift the acceptor end and disrupt the pairing with the antiterminator bulge, while stabilization of the 3-70 pair may enhance the pairing interaction due to a shift at the acceptor end in the opposite direction. This suggests that proper presentation of the tRNA acceptor end to the antiterminator bulge relies on the balance between tRNA structural rigidity and flexibility.

tRNA structural flexibility has been shown to be important during decoding and aminoacylation [35,36,37,38,39,40,41]. The conformational change in during decoding contributes to high-fidelity tRNA selection [42]. Our results support the idea that tRNA is a molecular spring [43] that plays an active role not only in translation, but also in tRNA-mediated antitermination of the T box regulatory system.

Protein synthesis is essential for cell survival; therefore, tRNAs have been subject to primary selective pressures from the translation machinery. In addition to the conserved tRNA elements that assist in maintenance of the canonical L-shape structure [44,45,46], some residues in tRNA may have coevolved with the anticodon sequence to tune each tRNA for uniform decoding activity during translation [18,47,48]. tRNA isoacceptors also must maintain both identity determinants and antideterminants to interact specifically with their cognate aaRS [49,50]. Each aa-tRNA is additionally tuned to compensate for the different thermodynamic contributions of each amino acid, and to achieve equivalent EF-Tu binding affinity [51,52]. Pre-tRNA tuning for RNase P recognition has also been reported [53]. Our study reveals an additional role for tRNA residues in tuning tRNA for optimal interaction with the cognate T box leader RNA.

Sequence variations between *C. acetobutylicum* tRNA^Ala^ (UGC) and *B. subtilis* tRNA^Ala^ (UGC) are located at positions that show less anticodon-dependent conservation among tRNA^Ala^ (UGC) from different organisms, including those that do not utilize the T box system [18]. tRNA sequence variations may be the result of different selective pressures from the translation machinery in each organism, that may be due to differences in genomic G+C content or environmental constraints; it is likely that T box leader RNAs have evolved to recognize those variations in its cognate tRNA. Alternatively, it is also possible that in organisms that utilize the T box riboswitch, the T box leader RNA may have imposed additional selective pressure onto the cognate tRNAs. The resulting variations are likely to occur at weakly conserved positions so that the tRNA retains the L-shape structure, uniform decoding efficiency during translation, and recognition by AlaRS, RNase P, and EF-Tu.
